# Identification of cancer stem cell-related biomarkers in intestinal-type and diffuse-type gastric cancer by stemness index and weighted correlation network analysis

**DOI:** 10.1186/s12967-020-02587-3

**Published:** 2020-11-07

**Authors:** Rui Guo, Aining Chu, Yuehua Gong

**Affiliations:** 1 Tumor Etiology and Screening Department of Cancer Institute and General Surgery, Liaoning Province, The First Hospital of China Medical University, No.155 NanjingBei Street, Heping District, Shenyang, 110001 P.R. China; 2 Key Laboratory of Cancer Etiology and Prevention in Liaoning Education Department, The First Hospital of China Medical University, Shenyang, 110001 China; 3 Key Laboratory of GI Cancer Etiology and Prevention in Liaoning Province, The First Hospital of China Medical University, Shenyang, 110001 China

**Keywords:** Diffuse-type gastric cancer, Intestinal-type gastric cancer, Cancer cell stemness, mRNAsi, WGCNA

## Abstract

**Background:**

Cancer stem cells (CSCs) play an important role in drug resistance, recurrence, and metastasis of tumors. Considering the heterogeneity of tumors, this study aimed to explore the key genes regulating stem cells in intestinal-type and diffuse-type gastric cancer.

**Methods:**

RNA-seq data and related clinical information were downloaded from The Cancer Genome Atlas (TCGA). WGCNA was used to clustered differentially expressed genes with similar expression profiles to form modules. Furtherly, based on the mRNA expression-based stemness index (mRNAsi), significant modules and key genes were identified. Next, the expression of key genes was further verified by the Oncomine database.

**Results:**

MRNAsi scores of GC were significantly higher than that of normal tissue. Additionally, mRNAsi scores of intestinal-type GC (IGC) were significantly higher than that of diffuse-type GC (DGC). WGCNA showed that the blue module of IGC and the brown module of DGC were both the most significantly associated with mRNAsi. We screened out 16 and 43 key genes for IGC and DGC and found that these genes were closely related, respectively. Functional analysis showed the relationship between the key genes confirmed in the Oncomine database and the fate of cells.

**Conclusions:**

In this study, 16 and 43 genes related to the characteristics of CSCs were identified in IGC and DGC, respectively. These genes were both associated with cell cycle, which could serve as therapeutic targets for the inhibition of stem cells from both types of GC.

## Background

As a common neoplasm worldwide, gastric cancer (GC) is the main cause of cancer-related deaths [1]. According to cancer statistics released by the International Agency for Research on Cancer (IARC), there were 952,000 new cases of GC worldwide in 2012, accounting for 68% of cancer patients, of which 723,000 die, about 88% of all cancer patients [2]. Approximately 90% of GC are adenocarcinoma originated from gastric mucosa. According to the Lauren classification, GC can be separated into the intestinal-type (IGC) and diffuse-type (DGC), which are significantly different in terms of tissue morphology, pathogenesis, biological behavior, prognosis, and survival [3, 4]. Therefore, an in-depth exploration of the molecular characteristics of IGC and DGC is of great significance for clinical diagnosis and treatment.

Recently, cancer stem cells (CSCs) have attracted more and more attention. And they are characterized as cells in the tumor which have the capacity of self-renewing and causing the heterogeneous cancer cell lineages. Many studies have shown that CSCs play a crucial role in the metastasis, differentiation, and drug-resistance of cancer [5–7]. At present, the recognition markers of hematologic tumor stem cells have been well known, but the research on solid tumor stem cells has not been very clear [8]. In recent years, CD44 has come to be gradually recognized as a marker for many solid tumor stem cells and CD44^+^ cells possess properties of radio- and chemo- resistance [10–14]. The combination of CD44 and epithelial cell adhesion molecule (Epcam) has also been found as putative gastric CSCs markers in GC [15]. However, further studies showed that the frequency of CD44 expression in IGC was significantly higher than that in DGC [16]. ALDH1, another marker of CSCs in several types of tumors, has been detected in DGC in 2012. And ALDH^+^ GC cells have strong tumorigenic and self-renewal ability. However, ALDH1 mRNA expression cannot be detected in IGC [17]. Some scholars suggested that the high heterogeneity of GC may be the main reason for the unidentified CSCs markers. Hence, exploring the differences between IGC and DGC from the perspective of stem cells is of clinical importance. Despite there has been increasing attention, the characteristics of CSCs in IGC and DGC remain to be further explored.

To better describe the characteristics of CSCs, *Malta *et al. compared the genetic maps of tumor cells and embryonic stem cells and proposed a new index, stem cell index. They analyzed a dataset of 99 stem/progenitor cells from the Progenitor Cell Biology Consortium (https://www.synapse.org/pcbc) and used a one-class logistic regression machine learning algorithm (OCLR) to train on different types of stem cells and progenitor cells, and thus got the stemness index. Furthermore, they applied the stemness index analyze the transcriptome of TCGA and obtained the mRNA expression-based stem index (mRNAsi) of different types of tumors including GC, with the value between -1 and 1, and the higher the value of mRNAsi, the stronger properties of cancer stem cells. The mRNAsi is considered as a quantitative indicator of CSCs to show how many tumor cells will be with the same properties as stem cells. Previous researches have also revealed that mRNAsi was associated with biological processes and tumor heterogeneity, which provided novel insights for further cancer research [18]. Based on the principle of the stemness index, many scholars have identified stem cell-associated key genes and possible signal pathways in bladder cancer and lung adenocarcinoma by using mRNAsi [19, 20]. This undoubtedly provides a novel direction for further research of tumor stem cells, yet the research of mRNAsi in GC has not been realized.

Given the close relationship between tumor stem cells and tumors, in this study, we explored the characteristics of GC histological subtype by the stemness index, and the WGCNA model was used to determine the most relevant module of mRNAsi index, with the key genes identified. Our study intends to explore markers associated with the stem cell characteristics of IGC and DGC, which would help clarify the biological characteristics and progression of GC subtypes and inform the future diagnosis and therapy of GC.

## Materials and methods

### Data collection and pre-processing

The RNA-seq data of 30 normal and 343 human gastric adenocarcinomas (GAC) specimens were downloaded from the TCGA database (https://portal.gdc.cancer.gov) in March 2020. The mRNAsi of normal and GAC specimens in TCGA were downloaded from previous studies [18]. When we made a comparison between normal and tumor samples, 1:4 under-sampling was used to balance the cases, and finally, 30 normal and 118 GAG (60 cases of IGC and 58 cases of DGC) were included [21]. In the comparison of mRNAsi and clinical features, 323 cases with complete information were chosen, including 139 cases of IGC and 58 cases of DGC. The RNA-seq data of every specimen was integrated by Perl language (https://www.perl.org), and gene names were converted into the corresponding gene symbols through the Ensemble database (https://asia.ensembol.org/index.html).

### Screening of differentially expressed genes (DEGs)

DEGs between tumor and normal tissues were identified by the limma package in R 3.6.1:false discovery rate (FDR) < 0.05, *P* < 0.01 and |log2-fold change| > 1.

### WGCNA

WGCNA (weighted gene co-expression network analysis) is a multiplex analysis method to cluster genes with similar expression patterns. The WGCNA package in R was utilized to build a co-expression network of DEGs [22]. First, the co-expression similarity matrix of genes was established by the average linkage method and Pearson's correlation matrices. And the soft thresholding parameter (β) was set to show the strong relations among genes. Then we performed topological overlap matrix (TOM) to measure the network connectivity of genes, sum up the adjacent genes for the network gene ratio and calculate the corresponding dissimilarity. As previously reported, through further analysis, DEGs were clustered with similar expression profiles to form modules, and the hierarchical clustering tree was constructed [19].

### Identification of significant module and key genes

Further, we chose significant modules related to mRNAsi, and genes in these modules were considered to be co-expression of genes related to CSC. First, gene significance (GS) was calculated, which reflected a linear regression between the gene expression and mRNAsi in each module. A cutoff value (< 0.5) was applied in the merge of highly similar modules. And then the modules that had the largest average GS were considered the most mRNAsi-related modules. After finding the most significant module, we used module membership (MM) to determine which gene in each module was highly related to mRNAsi, that is, to screen out key genes. The threshold values for the selection in the module of IGC or DGC were set: cor. GS > 0.5, cor. MM > 0.8; cor. GS > 0.8, cor. MM > 0.8, respectively.

### Functional enrichment analysis

Gene ontology (GO) functional annotation and Kyoto Encyclopedia of Genes and Genomes (KEGG) analysis were performed to study the biological functions of the key DEGs. And these analyses were conducted by the cluster profiler package in R [23]. *P*-value < 0.05 and FDR < 0.05 were chosen as the criteria in this section.

### Co-expression analysis and protein–protein interaction (PPI) network construction

R package corrplot was selected to estimate the Pearson correlation to illuminate the co-expression relationship of key genes at the transcriptional level [24]. The 11.0 version of STRING (https://www.string-db.org) was chosen to investigate and generate the PPI network among key genes.

### Oncomine database validation

Oncomine (https://www.oncomine.org) was investigated the mRNA expression of key genes between histological subtypes of GC and normal samples. The threshold was set as: gene rank = top10%; fold change = 2; *P*-value = 1E−4.

### Statistical analysis

The statistical analysis was managed by R language (Version 3.6.1) and SPSS (Version 21). The difference of mRNAsi scores between the normal and the tumor group was calculated using the Wilcox test and multivariate logistic regression analysis adjusted by age and sex. The Kruskal test analysis and multivariate logistic regression were selected to clarify the interrelationships between the scores of mRNAsi and clinical characteristics. The survival package was performed to evaluate the prognostic value of mRNAsi, a two-sided log-rank test, and multivariate Cox proportional hazards models adjusted for age, sex, stage, grade were employed to determine statistical significance. A *P*-value of no more than 0.05 was deemed statistically significant.

## Results

### Correlation between mRNAsi and clinical characteristics in GAC

**Fig. 1:**
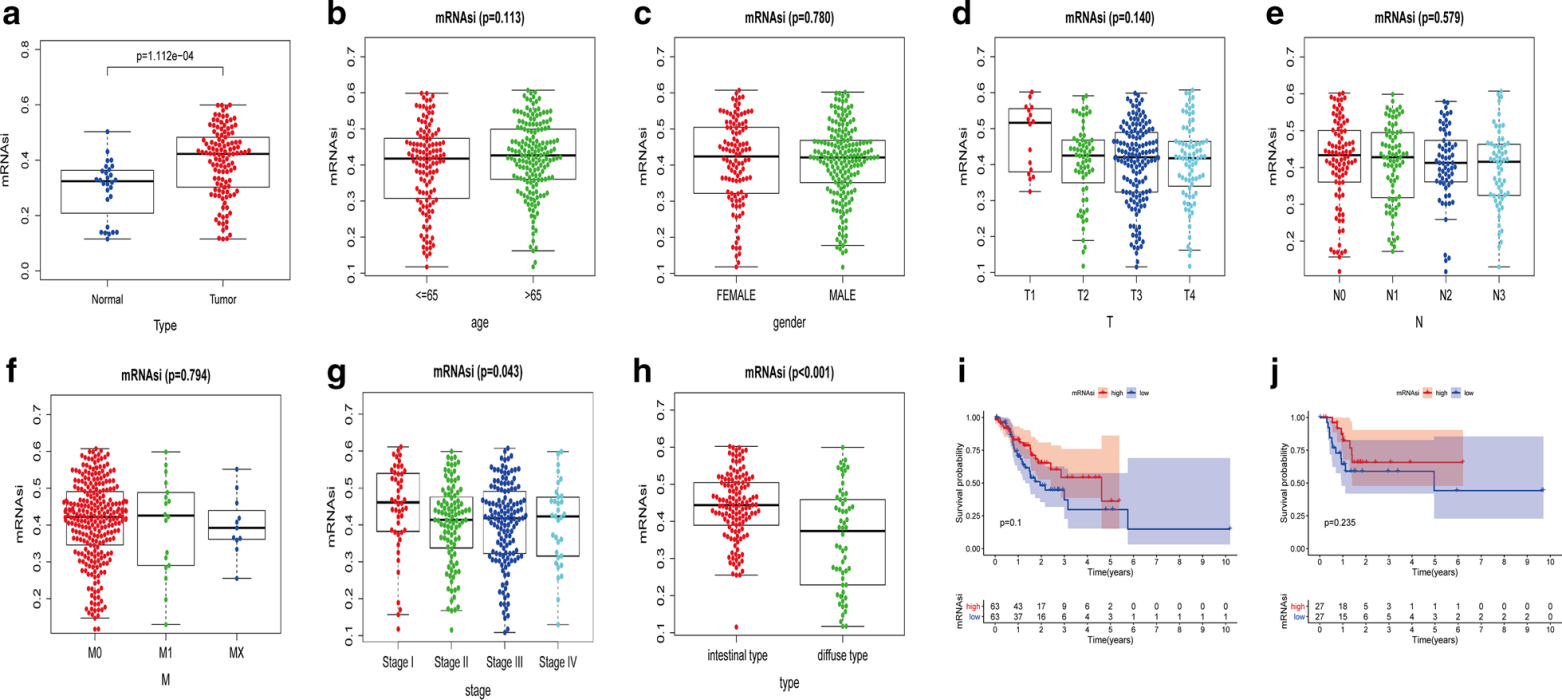
Correlation between mRNAsi and clinical characteristics in GAC. **a** Differences in mRNAsi between normal (30 samples) and GAC (118 samples) tissues. Comparison between mRNAsi expression level and clinical characteristics in GAC, including age (**b**), gender (**c**), TNM stage (**d−f**), tumor stage (**g**), and the type of GAC (**h**). **i** Kaplan–Meier survival curves of mRNAsi in IGC. **j** Kaplan–Meier survival curves of mRNAsi in DGC

As shown, when compared with normal samples, mRNAsi of tumor samples was significantly higher (Fig. 1a) and the result of multivariate logistic regression analysis, adjusted by age, gender, approved it with *P* < 0.001. Further, in terms of clinical features, 323 GAC patients were classified by age, gender, TNM stage, tumor stage, histopathological types, respectively; for which, mRNAsi was not associated with age (*P* = 0.133), gender (*P* = 0.780), T (*P* = 0.140), N (*P* = 0.579), M (*P* = 0.598) stage, but only had a declining trend with the improved tumor stage (Fig. 1b–g), and all the multivariate logistic regression results showed that the above analysis results were not statistically significant with *P* > 0.05. However, in terms of histological subtype, mRNAsi in the intestinal-type was significantly higher than that in diffuse-type (Fig. 1h) and multivariate logistic regression analysis approved it with *P* = 0.04. Finally, survival analysis indicated that the overall survival of patients with IGC and DGC made no sense (Fig. 1i, j) and multivariate Cox proportional hazards models also supportted this result with *P* > 0.05 of both.

### Screening of DEGs and stemness-related modules and key genes in IGC and DGC

**Fig. 2:**
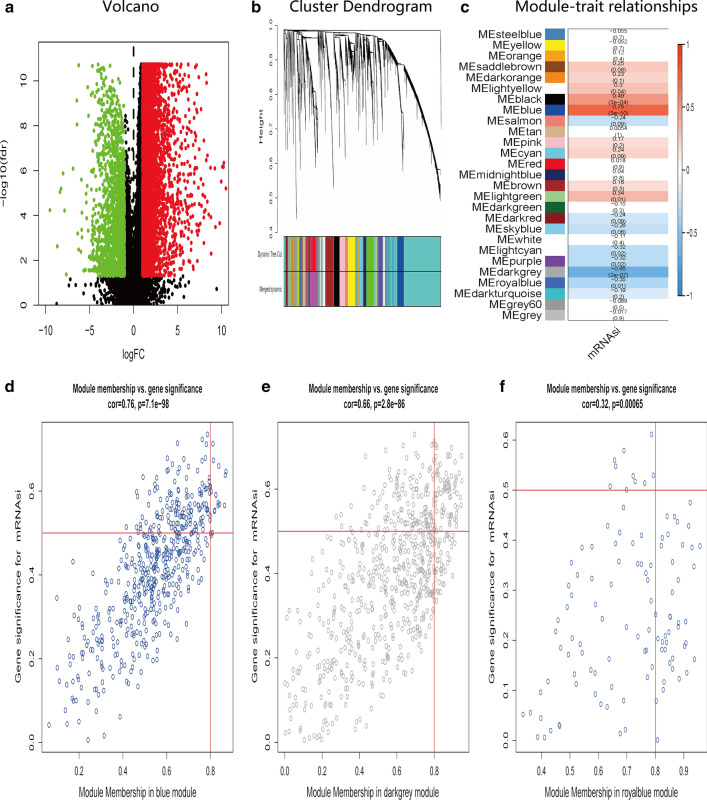
Screening of DEGs and stemness-related key modules in IGC. **a** DEGs: red indicated upregulated genes; green indicated downregulated genes and black indicated genes excluded by DEGs screening criteria. **b** WGCNA analysis of DEGs. Branches with different colors corresponding to different modules. **c** Correlation analysis of the modules and clinical traits with mRNAsi. *P*-values are shown. Scatter plot analysis of modules in the blue (**d**), darkgrey (**e**), and royalblue (**f**)

**Fig. 3:**
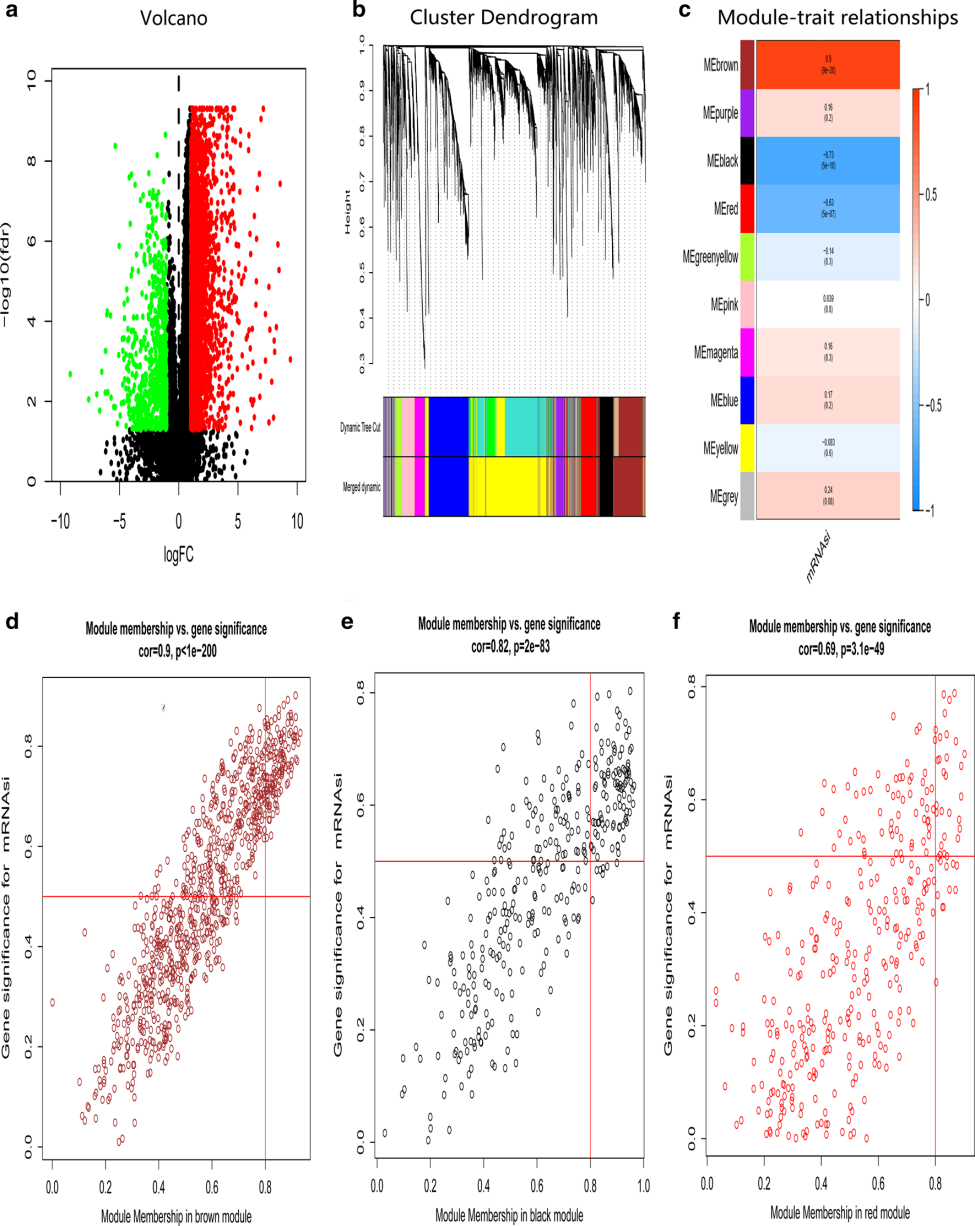
Screening of DEGs and stemness-related key modules in DGC. **a** DEGs: red indicated upregulated genes; green indicated downregulated genes and black indicated genes excluded by DEGs screening criteria. **b** WGCNA analysis of DEGs. Branches with different colors corresponding to different modules. **c** Correlation analysis of the modules and clinical traits with mRNAsi. *P*-values are shown. Scatter plot analysis of modules in the brown (**d**), black (**e**), and red (**f**) modules

The above results indicated that there may be specific DEGs that control the stemness of IGC and DGC, respectively. Therefore, we further filtered the significant modules and key genes with stemness properties. First, we compared the DEGs between IGC or DGC and normal samples, respectively. In IGC, 7596 DEGs were discerned, among which 5989 were over-expressed and 1607 were down-regulated (Fig. 2a and Additional file 1: Table S1); in DGC, 5424 DEGs were discerned, among which 4647 were up-regulated and 777 were down-regulated (Fig. 3a and Additional file 1: Table S2).

**Fig. 4:**
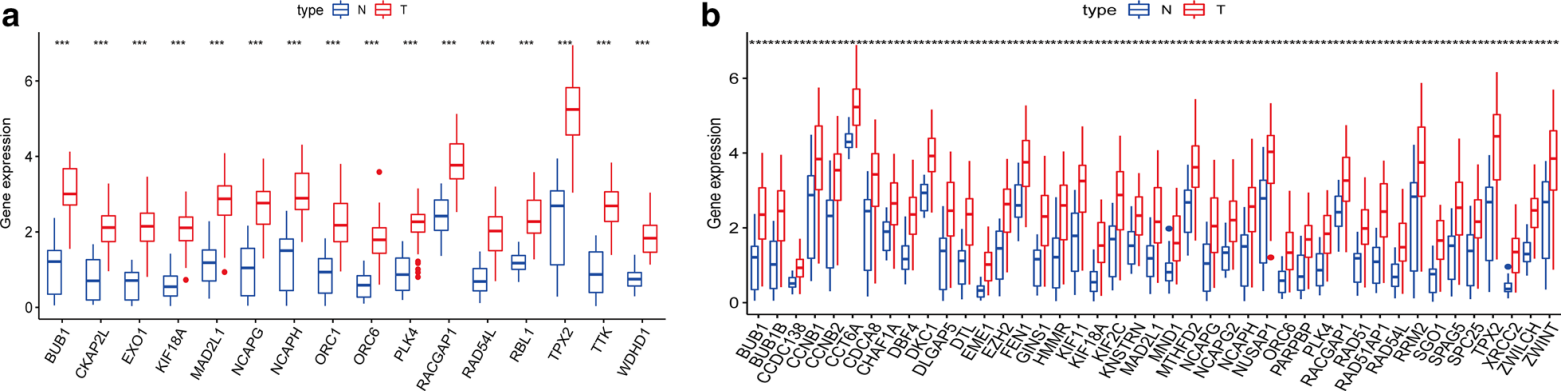
Expression of key genes related to mRNAsi. Expression levels of key genes in the blue module of intestinal-type (**a**) and brown module of diffuse-type (**b**) GC

Furthermore, WGCNA was used to construct a co-expression network of DEGs with the soft threshold β = 5 (scale-free R^2^ = 0.90) or β = 8 (scale-free R^2^ = 0.90) to ensure a scale-free network, and 27 and 10 gene modules with similar expression profiles in IGC and DGC were obtained for subsequent analysis (Fig. 2b, c; Fig. 3b, c). Then, we used MS as the overall gene expression level of the corresponding module to calculate the correlations between the modules and mRNAsi in IGC and DGC. Here, we noted that the blue module of intestinal-type with a correlation close to 0.8 and the brown module of diffuse-type with a correlation close to 1.0, both showed a positive correlation between stemness properties and gene expression. In addition, the dark-gray and royal-blue modules of IGC, and black and red modules of DGC exhibited relatively high negative correlations with mRNAsi. Thus, we chose the blue module of IGC and brown module of DGC as the modules of greatest interest. The thresholds of screening key genes in the significant modules were defined as cor.GS > 0.5, cor. MM > 0.8 and cor. GS > 0.8, cor. MM > 0.8 respectively. Finally, 16 stemness-related genes including ORC6, BUB1, NCAPH, ORC1, WDHD1, RACGAP1, CKAP2L, RBL1, KIF18A, TTK, TPX2, MAD2L1, NCAPG, RAD54L, EXO1, PLK4 have obtained for intestinal-type, as shown in Fig. 2d−f; 43 key genes for the diffuse-type, including DLGAP5, DTL, NCAPG2, KIF11, NCAPH, EZH2, ORC6, MTHFD2, BUB1, RAD54L, XRCC2, BUB1B, HMMR, KIF18A, KIF2C, NCAPG, EME1, PLK4, GINS1, PARPBP, SPAG5, ZWILCH, RAD51AP1, RACGAP1, CCDC138, TPX2, SPC25, MAD2L1, CCNB2, DKC1, KNSTRN, ZWINT, RAD51, NUSAP1, CHAF1A, SGO1, FEN1, CCNB1, RRM2, CDCA8, MND1, CCT6A, DBF4, as shown in Fig. 3d−f. Then we plotted their expression tendency in normal and tumor samples and discovered that the candidate genes were overexpressed in IGC and DGC, respectively (Fig. 4a, b). However, taking the intersection of key genes involved in IGC and DGC, BUB1, KIF18A, MAD2L1, NCAPG, RAD54L and PLK4 were left, which were more highly expressed in IGC.

### Validation of stemness-related key genes in the Oncomine database

**Fig. 5:**
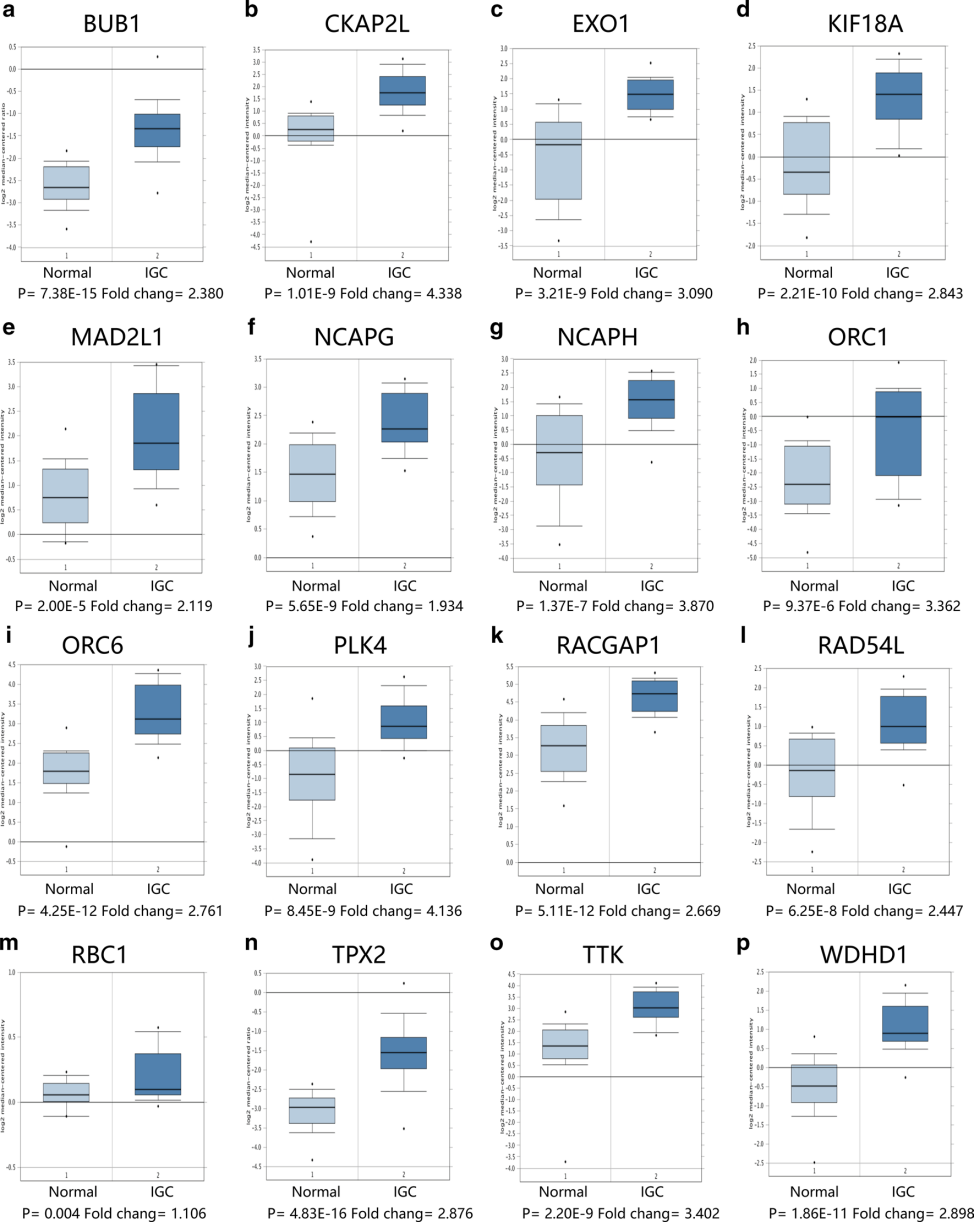
The mRNA expression of key genes in IGC in the Oncomine database

**Fig.6:**
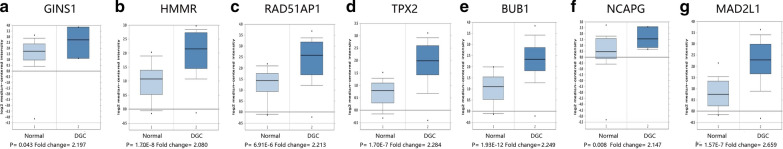
The mRNA expression of seven representative key genes in DGC in the Oncomine database

Next, the mRNAsi-related key genes were verified in the Oncomine database. As shown in Fig. 5, 16 genes of IGC were highly expressed. And except for CHAFIA, 42 key genes showed higher expression in DGC. Figure 6 showed 7 representative genes in DGC compared with normal tissues, and the remaining were shown in Additional file 2: Figure S1.

### The cellular functions and pathway analysis of stemness-related key genes

**Fig. 7:**
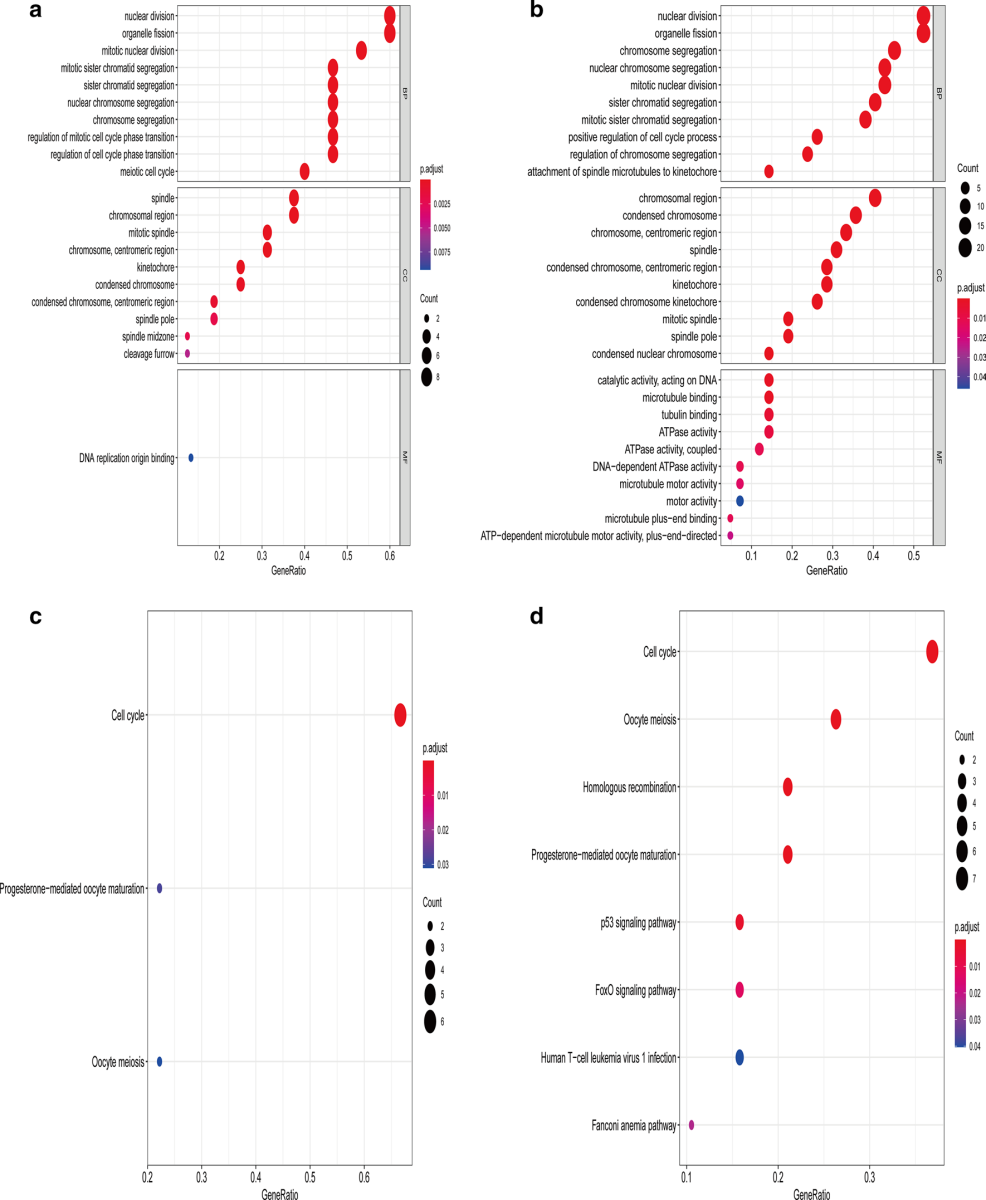
GO and KEGG pathway analysis of key genes. GO and KEGG analysis of the key genes in IGC (**a, c**) and DGC (**b, d**)

In IGC, the GO analysis results indicated that key genes participated in the biological process of nuclear division. The main cellular component manifested enrichment mainly at the spindle. The main molecular function enriched these genes in DNA replication origin binding, and the KEGG analysis demonstrated that the major pathway was cell cycle (Fig. 7a, c). In DGC, GO analysis results indicated that key genes participated in the biological process of nuclear division. The major cellular component suggested enrichment mainly in the chromosomal region. And the main molecular function was catalytic activity. KEGG analysis showed that the main pathway was also the cell cycle (Fig. 7b, d).

### Correlation between stemness-related key genes at transcription and protein levels

**Fig. 8:**
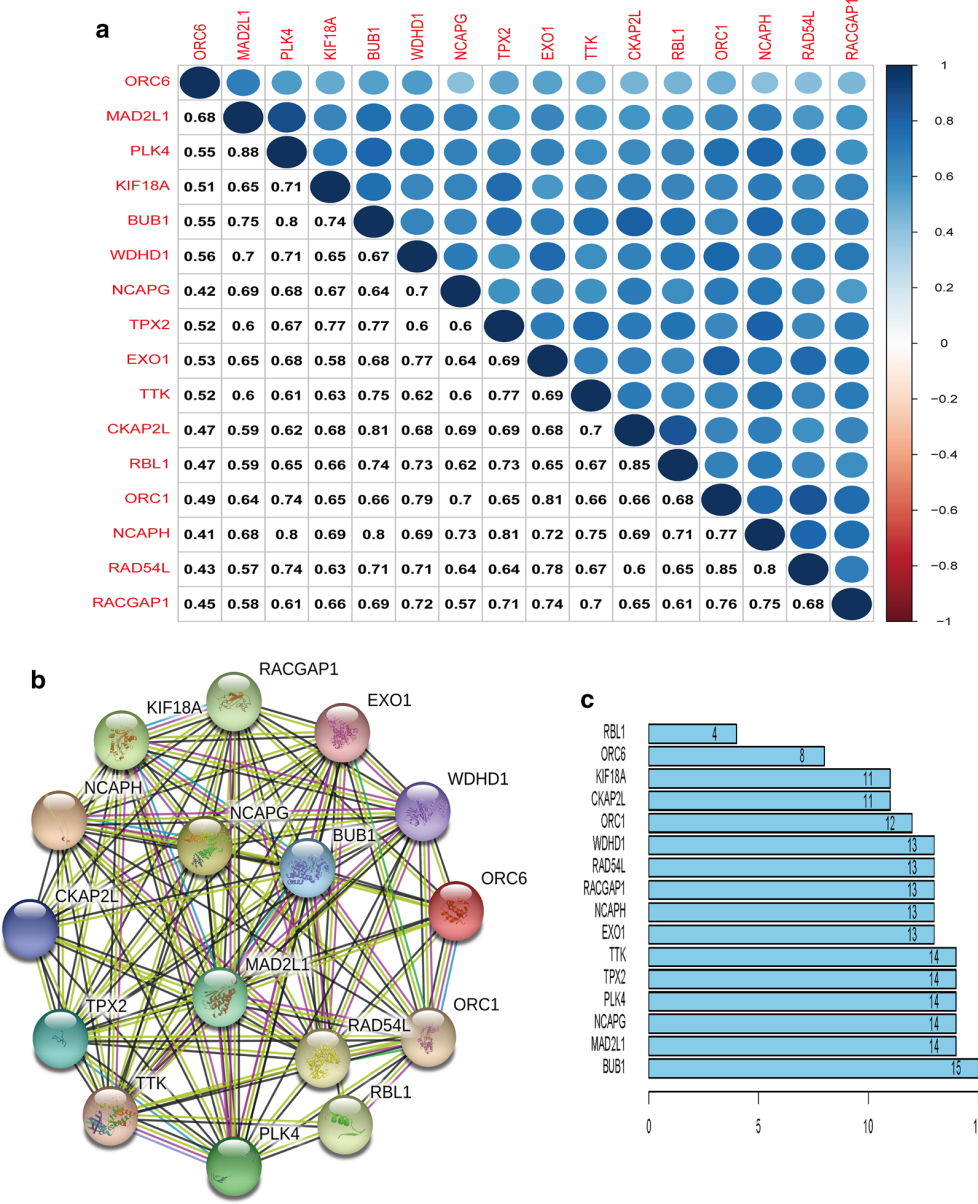
Correlation between key genes in IGC at transcriptional and protein levels. **a** Correlation between key genes at the transcriptional level. **b** The mutual PPIs of key genes. **c** The edge number of each key gene

**Fig. 9:**
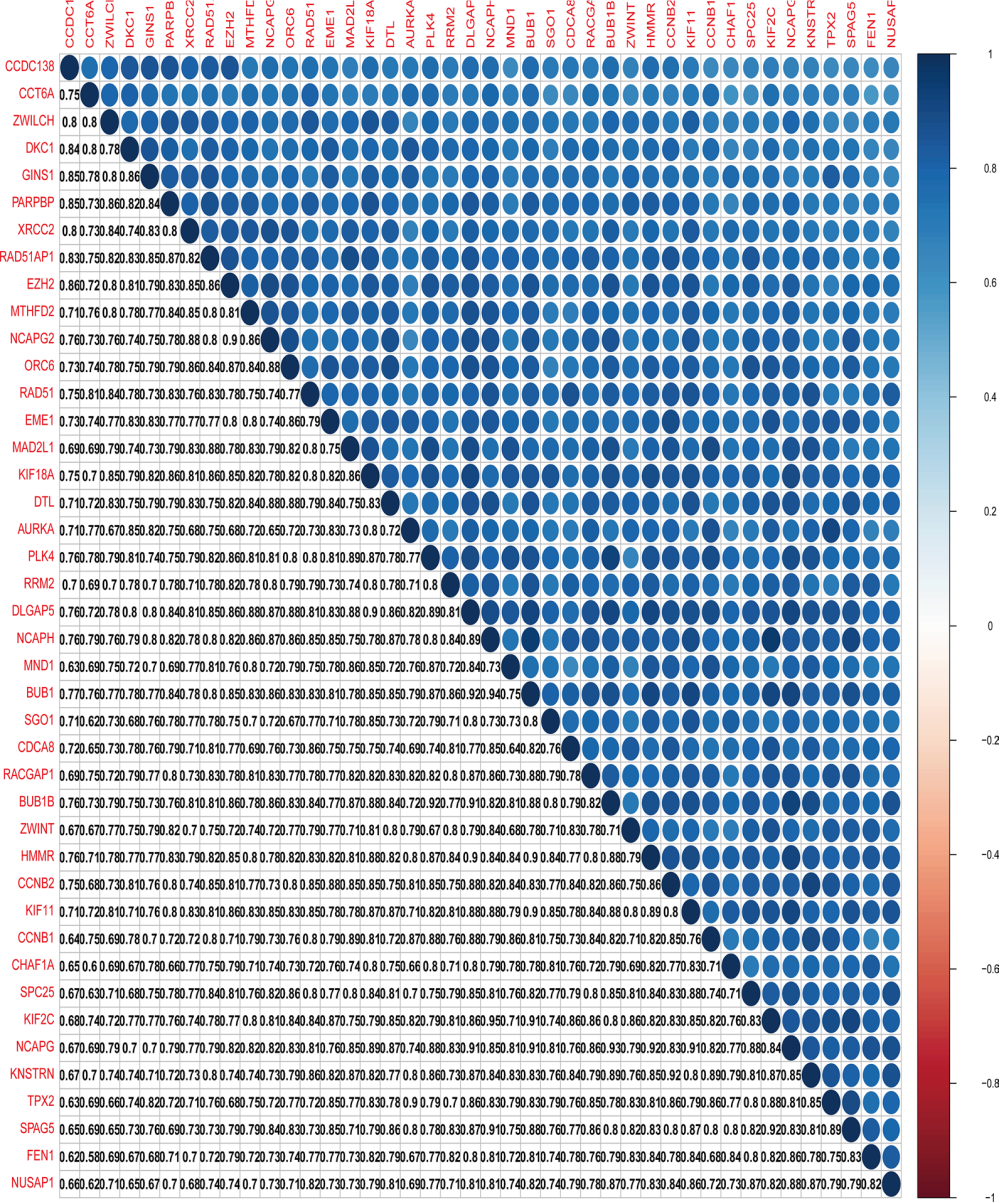
Correlation between key genes in DGC at the transcriptional level

**Fig. 10:**
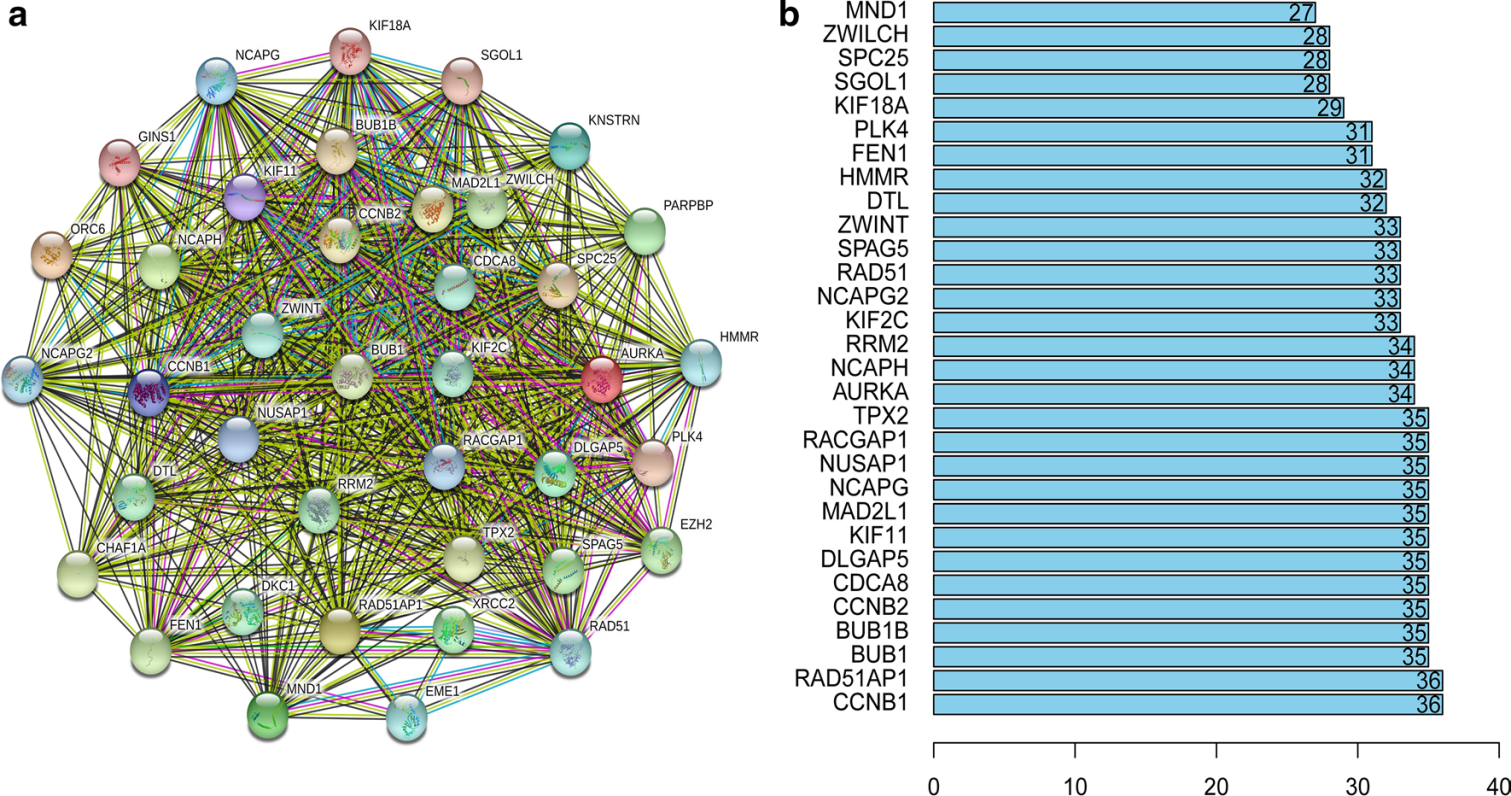
The mutual PPIs of key genes in DGC (**a**) and the edge number of each key gene (**b**)

We explored the interactive relation of the above key genes using Pearson correlation and found that the key genes in the blue module of IGC or the brown module of DGC had relatively strong correlation, with the minimum correlation coefficient of 0.41 and 0.58, and the correlation among genes was shown in Figs. 8a and  9, respectively. Next, we built the PPI network using the STRING online tool. As shown in Figs. 8b and  10a, the key genes of the two types of GC formed a close interaction relationship respectively. In IGC, the most crucial key protein was BUB1 (15 edges) (Fig. 8c). The most important key proteins in DGC were CCNB1 (37 edges) and RAD51AP1 (37 edges) (Fig. 10b) and BUB1 also had high connectivity.

## Discussions

GAC, as the main type of GC, can be classified into the diffuse and intestinal type. IGC occupies the majority with a better prognosis, while DGC is more malignant with a worse prognosis. Although numerous researches have spotted the diagnosis and treatment of these two types of GC, the molecular characteristics of them are still not clear. In this study, using mRNAsi, we screened out 16 genes related to stem cell characteristics of IGC and 43 genes of DGC. Further analysis showed that these key genes had strong interaction and had high co-expression relationship at the transcription and protein levels. These results suggested that key genes selected based on mRNAsi may indicate different molecular characteristics of IGC and DGC. Our study extended the knowledge of GC molecular characteristics and would provide new insight into the clinical treatment of GC histological subtypes.

We initially analyzed the relationship between clinical characteristics and mRNAsi scores in GAC, and the results showed that tumor samples had a higher stemness index when compared with normal samples, which was consistent with former studies [25]. Meanwhile, in terms of clinical features, mRNAsi was not correlated with age, gender, TNM stages, and tumor stages. However, there were significant differences between IGC and DGC. In survival analysis, the mRNAsi index in IGC and DGC had no statistical significance with overall survival. Since mRNAsi had a close relationship with the histology subtype, we speculated that there may be some key genes differentially expressed that control the stemness properties of different subtypes of GC, respectively.

Furthermore, we used WGCNA to distinguish significant modules related to mRNAsi and identify stemness-related key genes. In IGC and DGC, the blue module and the brown module showed significant positive correlations with mRNAsi respectively, indicating that the key genes in these modules had higher stem cell characteristics. Based on GS and MM, 16 and 43 stemness-associated genes were obtained from IGC and DGC, respectively, and in two types of GC, these key genes were highly expressed. Further functional analysis revealed that the key genes in IGC and DGC were both primarily concentrated in the cell cycle pathway. The cell cycle pathway plays a critical role in the development of GC, which is not only associated with the proliferation of GC cells but also related to the prognosis of the tumor. For example, cyclin D1 was one of the biomarkers of poor prognosis in GC patients [28]. And the differential expression of cell cycle-related genes may also be related to the different development of IGC and DGC.

Moreover, among the key genes obtained above, BUB1 was highly expressed in both IGC and DGC and has higher connectivity in PPI network, but it was with highly expression in IGC. As a common candidate key gene, BUB1 had a mitotic serine/threonine kinase checkpoint that was up-regulated in numerous cancers and associated with tumorigenesis, proliferation, and metastasis [29–31]. In the cell cycle, BUB1 gradually accumulated and peaked at G2 / M phases, playing a key part in cell division [32, 33]. Inhibition of BUB1 can further prevent tumor proliferation and increase cell apoptosis by regulating TGF β / Smad signaling pathway [34]. The different expression levels of BUB1 in IGC and DGC may provide new ideas for the diagnosis and treatment of GC.

The Oncomine validation indicated that all key genes were highly expressed in IGC, among which PLK4 is a serine/threonine-protein kinase that regulates centriole duplication. It has been reported that its deregulation could cause centrosome number abnormalities, mitotic defects, chromosomal instability, and, consequently, tumorigenesis. And PLK4 has emerged as a therapeutic target for the treatment of multiple cancers [41]. This demonstrated that PLK4 may also be a possible therapeutic target for IGC. CKAL2L, also known as radial fiber and mitotic spindle, is a mitotic spindle protein-coding gene located on 2q14.1. Yumoto et al. have demonstrated the crucial role of CKAL2L in the cell-cycle progression of neural progenitors and mitotic spindle formation [42]. Meanwhile, it has been found that could act as an original prognostic biomarker and therapeutic target of hepatocellular carcinoma [43]. Therefore, this gene may play a similar role in IGC. In DGC, MAD2L1, RAD51AP1, TPX2, NCAPG, GINS1, HMMR, BUB1 were highly expressed of 43 stemness-related key genes, among which MAD2L1 was an important component of the mitosis checkpoint protein. Previous studies showed that MAD2L1 was up-regulated as a proto-oncogene which could promote cell proliferation in GC [37]. RAD51AP1 was a key protein in homologous recombination, and its up-regulation in intrahepatic cholangiocarcinoma and lymphoma could promote the development of cancer [38, 39]. Therefore, this gene may also contribute to the development of DGC. TPX2, a microtubule-associated protein, was associated with the malignant behavior of GC and the overall survival in patients with GC [40]. In addition, HMRR (hyaluronic acid-mediated motor receptor) was closely related to tumor recurrence and could induce epithelial-mesenchymal transition, therefore promoting the characteristics of GC stem cells. To make these identified genes in translational research, Power Analysis and Sample Size Software (PASS) version 11 (NCSS, LLC, Kaysville, Utah, United States) would help estimate the sample size. In summary, our studies suggested that the key genes in the brown module may be associated with the development of the above two types of GC, respectively.

There are some limitations to this article: First of all, although we used the under-sampling method to balance the sample, the actual sample equilibrium will make the results more reliable. Next, this study was a retrospective study based on TCGA network database, and the PPI network among key genes was explored with the STRING database, since the data contained in the database was generally not examined by a human curator, the conclusion and the relationship among genes need further biological verification. Finally, due to the limitation of sample size, the correlation between stem cell index and some clinical characteristics, such as survival, may not be well explored and needs to be further tested in a larger sample population.

In conclusion, we identified genes that maintained the characteristics of IGC and DGC. These genes could become therapeutic targets to inhibit the properties of both stem cells. However, our conclusions were based on retrospective data, so further biological studies are in need to verify these findings.

## Conclusions

Taken together, 16 and 43 genes correlated to the characteristics of CSCs were identified in IGC and DGC, respectively. These genes were related to cell cycle, which may serve as therapeutic targets for the inhibition of the stem cells from both types of GC. However, our conclusions derived from bioinformatic analysis still need further basic studies to validate.

## Supplementary information

**Additional file 1: Table S1.** The DEGs between the IGC and normal tissues. **Table S2.** The DEGs between the DGC and normal tissues.

**Additional file 2: Figure S1.** Expression validation of 36 stemness-related key genes expression in DGC.

## Data Availability

The datasets supporting the conclusions of this article were available in the TCGA (https://portal.gdc.cancer.gov), Oncomine (https://www.oncomine.org). The datasets supporting the conclusions of this article are included within the article and its Additional files.

## Abbreviations

GC: Gastric cancer
IGC: Intestinal-type gastric cancer
DGC: Diffuse-type gastric cancer
GAC: Gastric adenocarcinoma cancer
PPI: Protein–protein interaction
IARC: International Agency for Research on Cancer
CSCs: Cancer sten cells
mDNAsi: DNA methylation-based stemness index
mRNAsi: MRNA expression-based stemness index
TCGA: The Cancer Genome Altas
WGCNA: Weighted gene co-expression network analysis
DEGs: Differentially expressed genes
GS: Gene significance
MM: Module eigengenes
PCA: Principal component analysis
GO: Gene ontology
KEGG: Kyoto Encyclopedia of Genes and Genomes
STRING: Search Tool for the Retrieval of Interacting Genes
PPI: Protein–protein interaction
EpCAM: Epithelial cell adhesion molecule
FDR: False discovery rate
HR: Homologous recombination
TRAIL: Inhibiting apoptosis-inducing ligand
HMRR: Hyaluronic acid-mediated motor receptor
